# Supplementary material for: In vivo imaging of invasive aspergillosis with 18F-fluorodeoxysorbitol positron emission tomography
Source: Nat Commun. 2022 Apr 8;13:1926. doi: 10.1038/s41467-022-29553-5 (PMC8993802; doi:10.1038/s41467-022-29553-5)
Supplement: Supplementary file 1 — Supplementary Information [file 41467_2022_29553_MOESM1_ESM.pdf]

Supplementary Information for

In vivo imaging of invasive aspergillosis with ^{18}F-fluorodeoxysorbitol positron emission tomography

Dong-Yeon Kim[3,6§], Ayoung Pyo[1,4§], Sehyeon Ji[5], Sung-Hwan You[2,6], Seong Eun Kim[5], Daejin Lim[7], Heejung Kim[8], Kyung-Hwa Lee[9], Se-Jeong Oh[10], Ye-rim Jung[1,2], Uh Jin Kim[5], Subin Jeon[1], Seong Young Kwon[1,2], Sae-Ryung Kang[1,2], Hyang Burm Lee[12], Hoon Hyun[13], So-Young Kim[2,6], Kyung-Sub Moon[10], Sunwoo Lee[11], Seung Ji Kang[5*], Jung-Joon Min[1,2,6*]

[1]*Innovation Center for Molecular Probe Development, Department of Nuclear Medicine, Chonnam National University Medical School and Hwasun Hospital, Hwasun, Korea*
[2]*Institute for Molecular Imaging and Theranostics, Chonnam National University Medical School, Hwasun, Korea*
[3]*College of Pharmacy and Research Institute of Pharmaceutical Science, Gyeongsang National University, Jinju, Korea*
[4]*Accelerator & RI Development Team, Korea Atomic Energy Research Institute, Jeongeup, Korea*
[5]*Department of Internal Medicine, Chonnam National University Medical School, Hwasun, Korea*
[6]*CNCure Biotech, Hwasun, Korea*
[7]*Division of Biomedical Convergence, College of Biomedical Science, Kangwon National University, Chuncheon, Korea*
[8]*Korea Radioisotope Center for Pharmaceuticals, Korea Institute of Radiological & Medical Sciences, Seoul, Korea*
[9]*Department of Pathology, Chonnam National University Medical School, Hwasun, Korea*
[10]*Department of Neurosurgery, Chonnam National University Medical School, Hwasun, Korea*
[11]*Department of Chemistry, Chonnam National University, Gwangju, Korea*
[12]*Department of Agricultural Biological Chemistry, Chonnam National University, Gwangju, Korea*
[13]*Department of Biomedical Sciences, Chonnam National University Medical School, Hwasun, Korea*

Address correspondence to: **Jung-Joon Min** (jjmin@jnu.ac.kr) and **Seung Ji Kang** (sseungi@gmail.com)

Jung-Joon Min, MD, PhD
Department of Nuclear Medicine, Chonnam National University Medical School
160 Ilsimri, Hwasun, Jeonnam 58128, Republic of Korea
Phone: 82-61-379-2876
Fax: 82-61-379-2875
E-mail: jjmin@jnu.ac.kr

Seung Ji Kang, MD, PhD
Department of Internal Medicine, Chonnam National University Medical School
160 Ilsimri, Hwasun, Jeonnam 58128, Republic of Korea
Phone: 82-61-379-7608
Fax: 82-61-379-7628
E-mail: sseungi@gmail.com

[§]These authors contributed equally to this work.

Table of Contents

Information	Page
Cellular uptake of ^{18}F-FDS by *A. fumigatus*, *R. arrhizus*, *C. albicans*, *S. aureus* (−), and *E. coli* (+) cells (Supplementary Figure 1)	S3
Confocal microscopy demonstrates live binding of sorbitol-ZW800-1 to different growth stages of *A. fumigatus* (Supplementary Figure 2)	S4
MicroPET, CT, and PET/CT fusion images of ^{18}F-FDS in mice with *A. fumigatus*-infected myositis (Supplementary Figure 3)	S5
A. fumigatus conidia to organ uptake ratio of ^{18}F-FDS in mice with *A. fumigatus*-infected myositis (Supplementary Figure 4)	S6
Pathological examination of a mouse with *A. fumigatus*-infected myositis (Supplementary Figure 5)	S7
Pathological examination of a mouse with *S. aureus*-infected lung tissue (Supplementary Figure 6)	S8
MicroPET, CT, and PET/CT fusion images of ^{18}F-FDS in mice with *A. fumigatus* lung infection (Supplementary Figure 7)	S9
Autoradiographic image of ^{18}F-FDS and pathological examination of *A. fumigatus*-infected lung tissue (Supplementary Figure 8)	S10
MicroPET, CT, and PET/CT fusion images of ^{18}F-FDS in mice with *S. aureus*-infected lungs (Supplementary Figure 9)	S11
MicroPET, CT, and PET/CT fusion images of ^{18}F-FDG in mice with *S. aureus* lung infection (Supplementary Figure 10)	S12
MicroPET/CT fusion images of ^{18}F-FDG in mice with *A. fumigatus* lung infection (Supplementary Figure 11)	S13

A. fumigatus-infected lung tissue to background (normal muscle of hind leg) uptake ratios of ^{18}F-FDS (2 h) and ^{18}F-FDG (1 h) post-injection (Supplementary Figure 12)	S14
MicroPET/CT fusion images of ^{18}F-FDS and ^{18}F-FDG in mice with B16F10 lung metastases (Supplementary Figure 13)	S15
MicroPET, CT, and PET/CT fusion images of ^{18}F-FDS and ^{18}F-FDG in mice with LPS induced lung inflammation (Supplementary Figure 14)	S16
Pathological examination of a mouse with LPS-induced acute lung injury (Supplementary Figure 15)	S17
MicroPET/CT fusion images of ^{18}F-FDS in mice with *A. fumigatus* brain infection at 48 h (Supplementary Figure 16)	S18
Body weights of *A. fumigatus*-infected mice before and after treatment with voriconazole or fluconazole for up to 20 days (Supplementary Figure 17)	S19

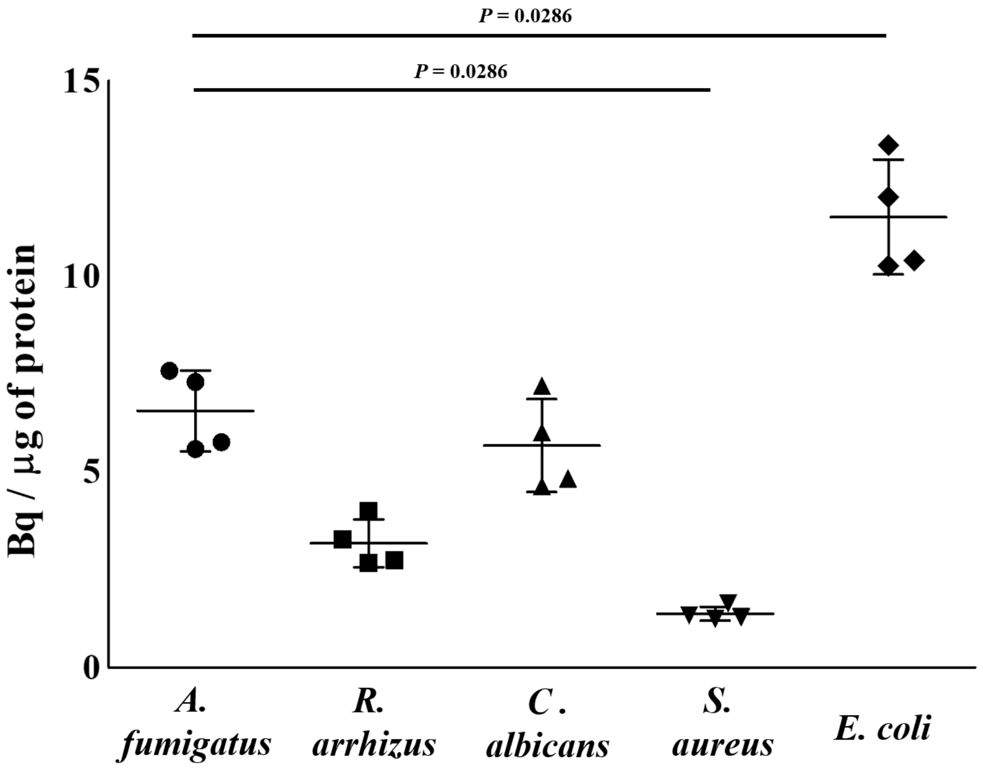

Supplementary Figure 1. Cellular uptake of ^{18}F-FDS by *A. fumigatus*, *R. arrhizus*, *C. albicans*, *S. aureus* (−), and *E. coli* (+) cells 1 h after treatment with ^{18}F-FDS. Data are expressed as the mean of absolute accumulation activity (Bq) ± SD (normalized by protein of 1 × 10^6 cells) of four replicate experiments ($^*P < 0.05$). Statistical significance was calculated using two-tailed Mann-Whitney U tests.

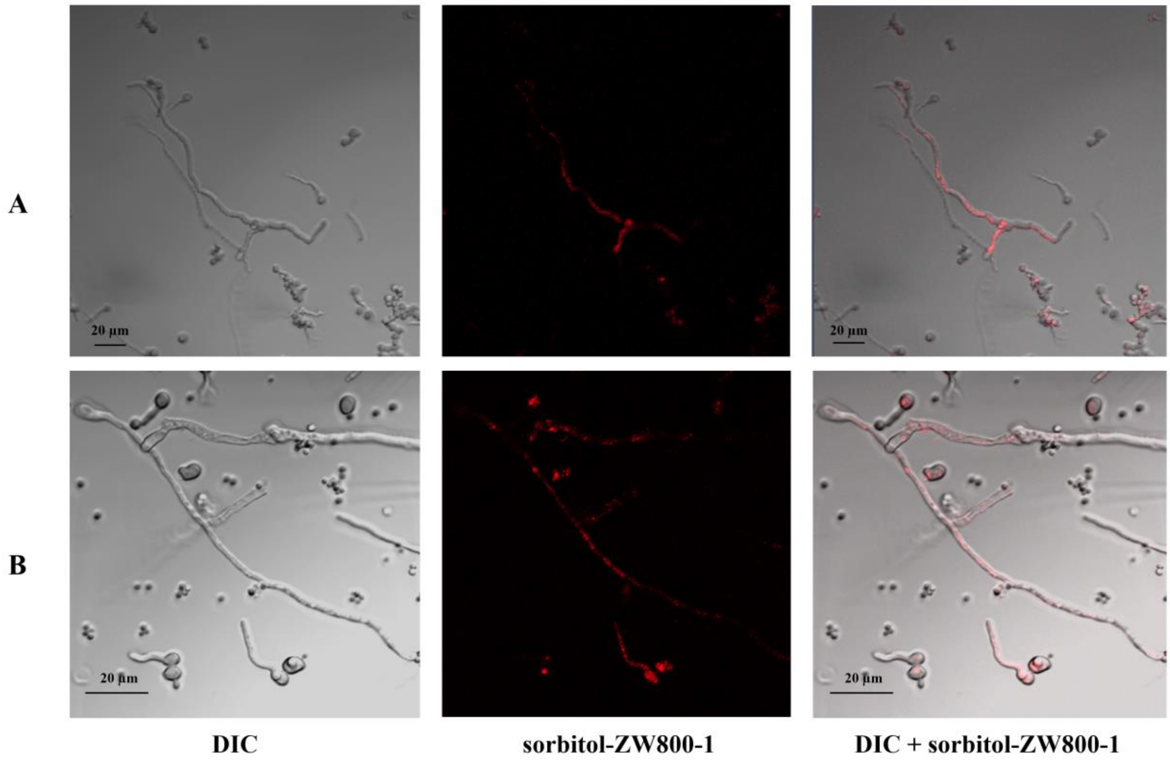

Supplementary Figure 2. Confocal microscopy demonstrates live binding of sorbitol-ZW800-1 to different growth stages of *A. fumigatus*. *A. fumigatus* was cultured on potato dextrose agar plates for 5–7 days at 30°C and colonies were harvested using saline containing 0.1% (v/v) Tween 20. The suspensions were vortexed to release the conidia and filtered through a 40 μm cell strainer to remove clumps and hyphae. Conidia were counted using a hemocytometer, and 1×10^7 *A. fumigatus* conidia and 50 μM of sorbitol-ZW800-1 were co-cultured for up to 48 h in 1 mL of YPD broth in 24-well plates at 37°C without agitation. Co-cultured *A. fumigatus* were observed at 20 and 48 h using laser scanning confocal microscopy (LSM 800, ZEISS, Germany). Before microscopic examination, cultured *A. fumigatus* were washed twice, re-suspended in PBS, and transferred to a confocal dish. Fluorescence of ZW800-1 (red) was obtained using laser filter excitation at 561 nm and emission at 650–750 nm. Image acquisition and analysis were performed using ZEN 2.6, blue edition (ZEISS, Germany). Merged differential interference contrast (DIC) and laser scanning images show more sorbitol accumulated in germinated *A. fumigatus* than in conidia. (A) Twenty hours of incubation × 200, n = 3; (B) forty-eight hours incubation × 400, n = 3.

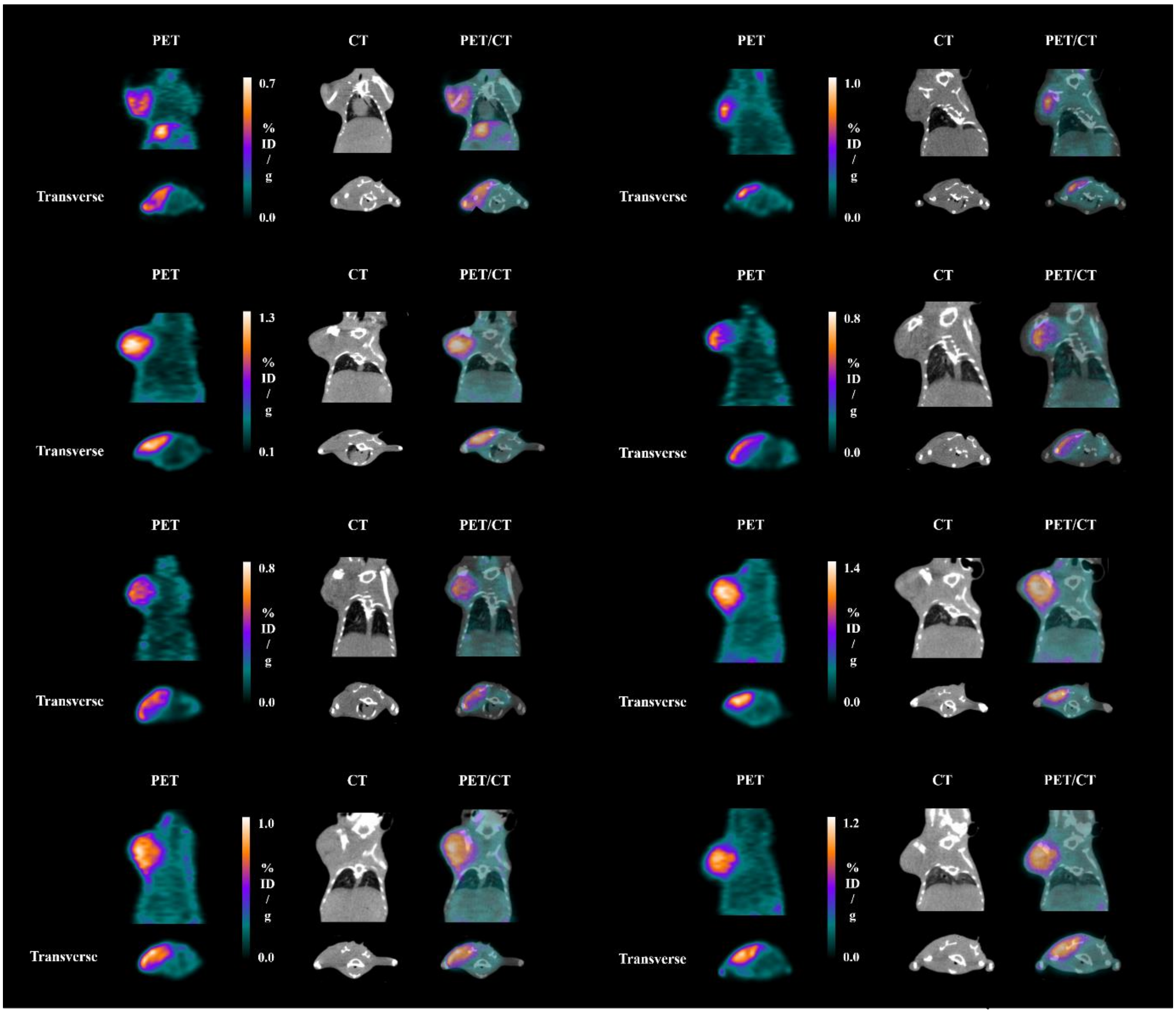

Supplementary Figure 3. MicroPET, CT, and PET/CT fusion images of ^{18}F-FDS 2 h after injection (7.4 MBq) in mice with *A. fumigatus*-infected myositis.

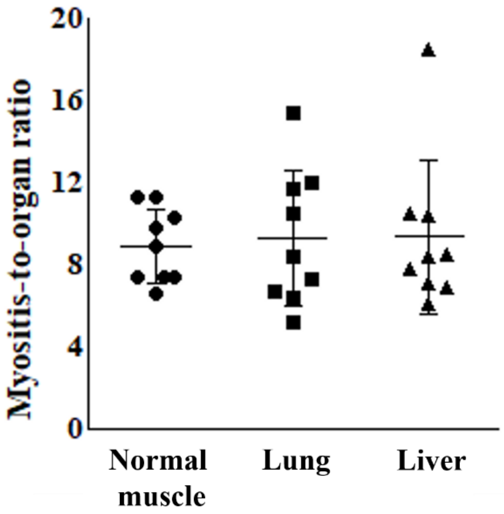

Supplementary Figure 4. *A. fumigatus* conidia to organ uptake ratio of ^{18}F-FDS in mice with *A. fumigatus*-infected myositis 2 h after injection (7.4 MBq, n = 9, each, Data are expressed as the mean ± SD).

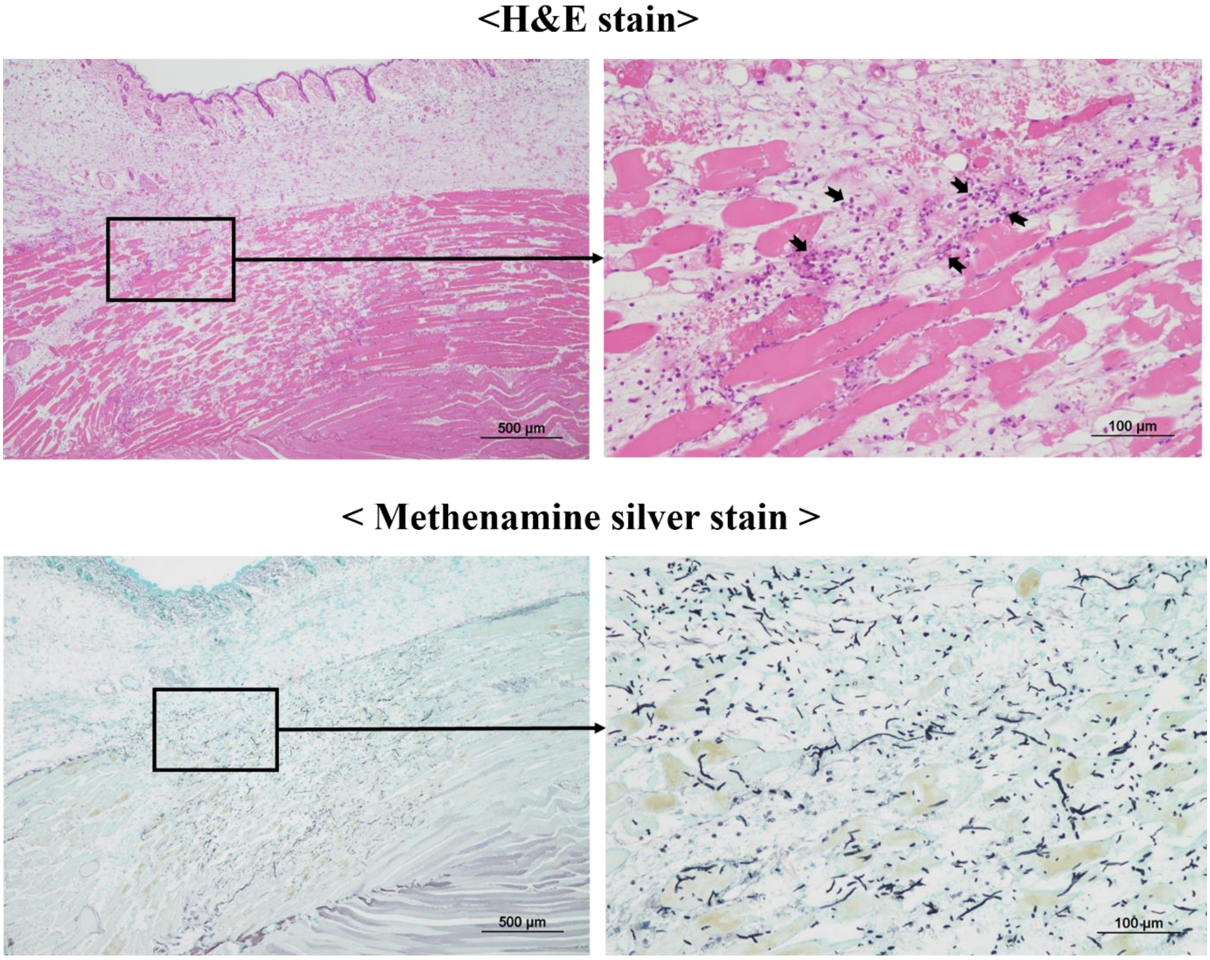

Supplementary Figure 5. Pathological examination of a mouse with *A. fumigatus*-infected myositis (n = 4). (Left upper) Hematoxylin and eosin (H&E) staining showing the destruction of muscle fibers and infiltration of inflammatory cells into muscle fibers (original magnification × 40). (Right upper). Magnified view of the box in the left upper figure (original magnification × 200). (Left lower) Methenamine silver staining showing scattered hyphae of *A. fumigatus* infiltrating into muscle fibers (original magnification × 40). (Right lower) Magnified view of the box in the left lower figure (original magnification × 200).

<H&E stain>

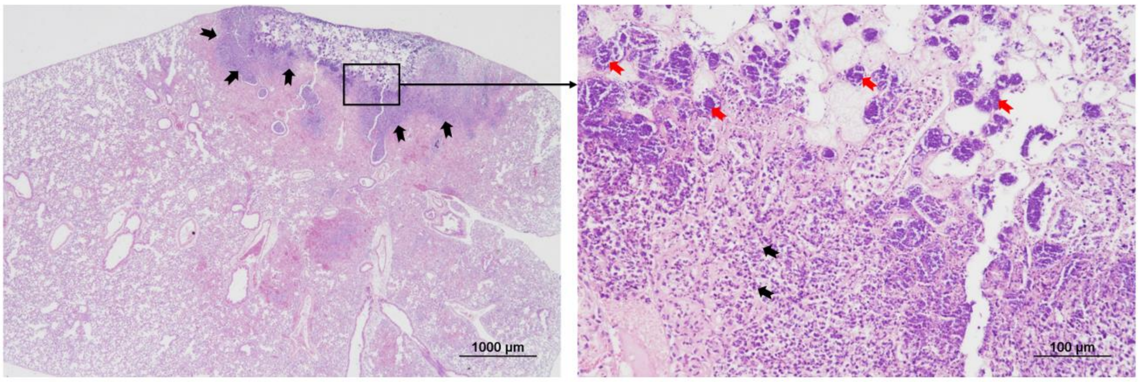

Supplementary Figure 6. Pathological examination of a mouse with *S. aureus*-infected lung tissue (n = 4). (Left side) Hematoxylin and eosin (H&E) staining, showing abscess formation and massive infiltration of inflammatory cells (arrows, original magnification × 20). (Right side) Magnification of the box in the left figure, showing clustered *S. aureus* (red arrows) and inflammatory cells (black arrows) (original magnification × 200).

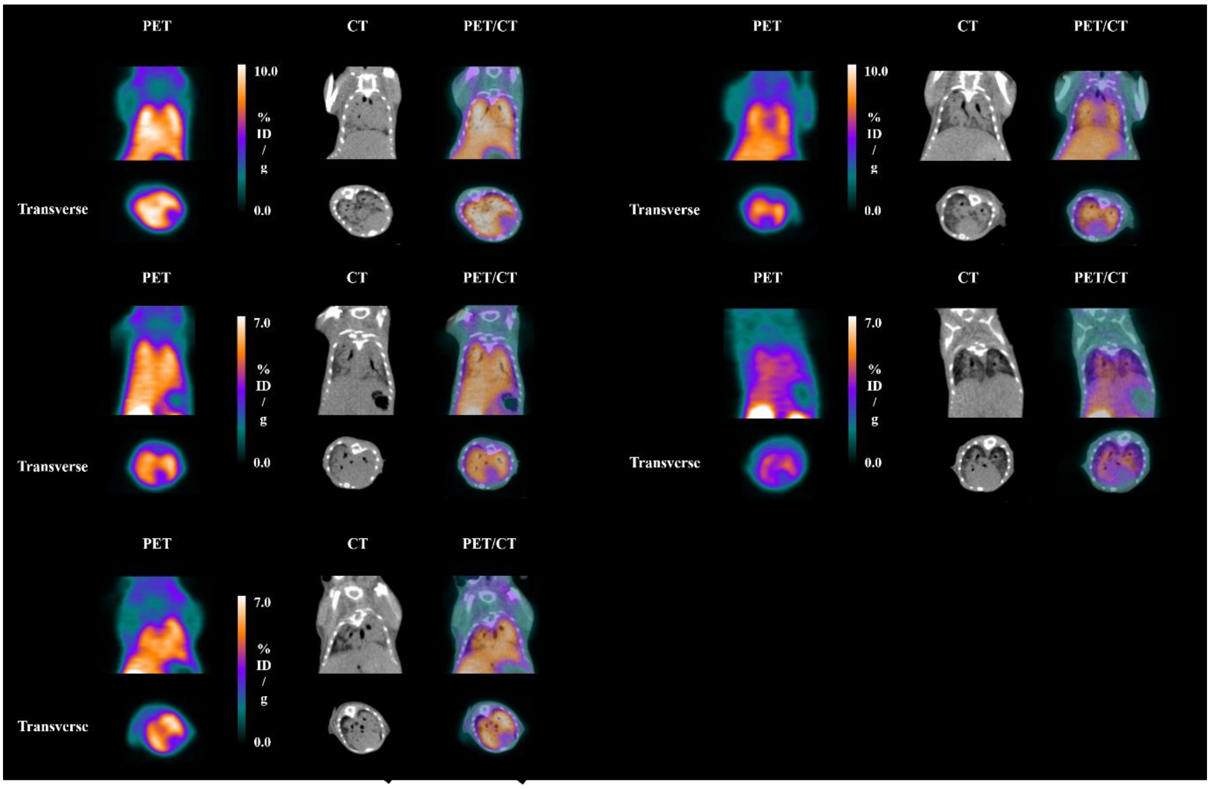

Supplementary Figure 7. MicroPET, CT, and PET/CT fusion images of ^{18}F-FDS 2 h after injection (7.4 MBq) in mice with *A. fumigatus* lung infection.

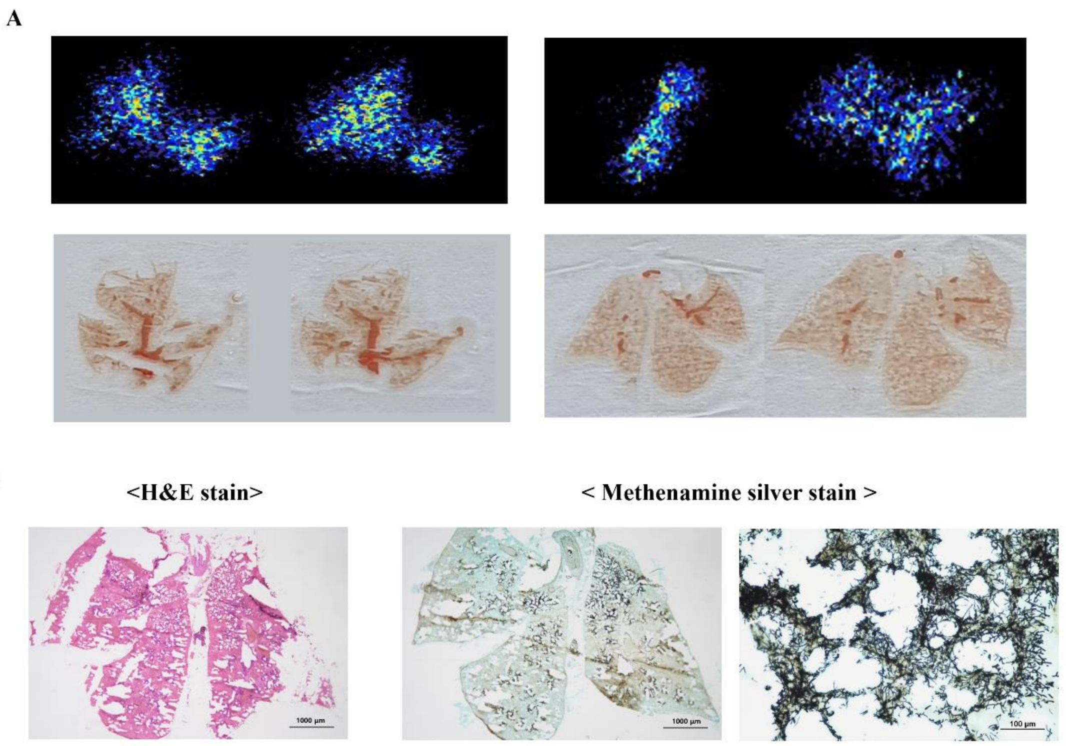

Supplementary Figure 8. (A) Autoradiographic image of ^{18}F-FDS and photograph of frozen *A. fumigatus*-infected lung sections. Mice with *A. fumigatus*-infected lungs were intravenously injected with 111 MBq of ^{18}F-FDS. The mice were sacrificed and their lungs were removed, and 25 μm frozen sections of lung were cut using a cryostat. (B) Pathological examination of *A. fumigatus*-infected lung tissue (n = 10).

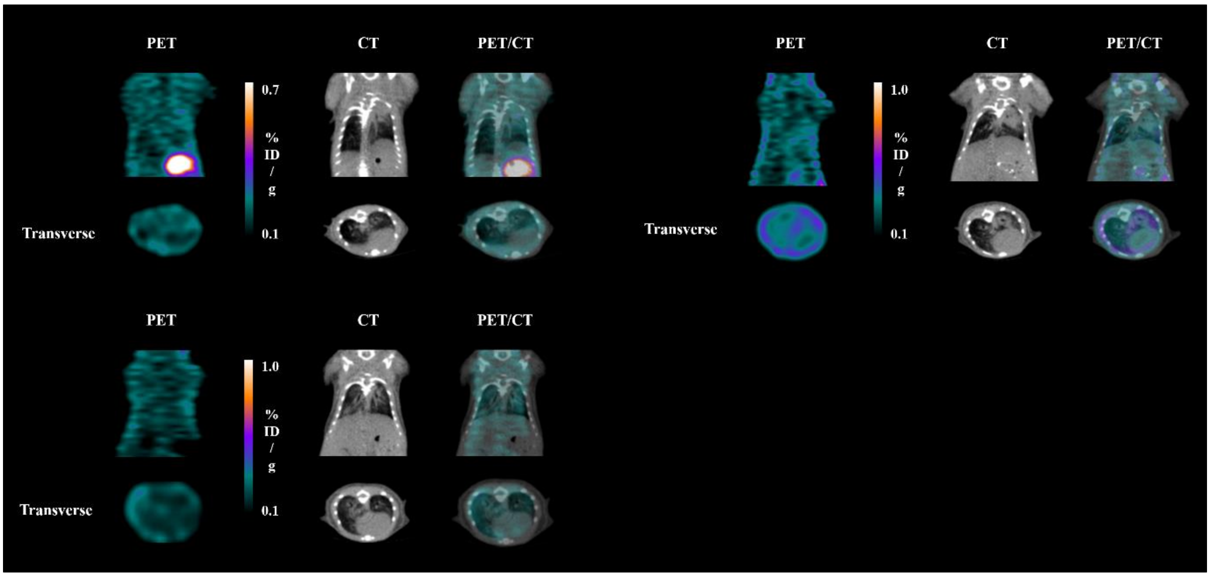

Supplementary Figure 9. MicroPET, CT, and PET/CT fusion images of ^{18}F-FDS in mice with *S. aureus*-infected lungs 2 h after injection of ^{18}F-FDS (7.4 MBq).

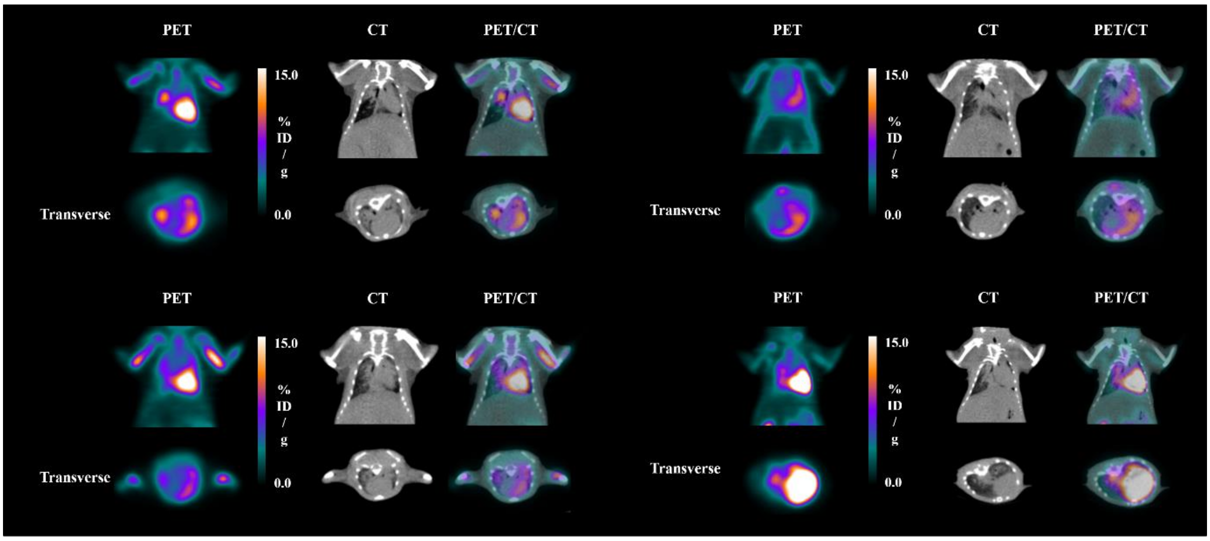

Supplementary Figure 10. MicroPET, CT, and PET/CT fusion images of ^{18}F-FDG in mice with *S. aureus* lung infection 1 h after injection of ^{18}F-FDG (7.4 MBq).

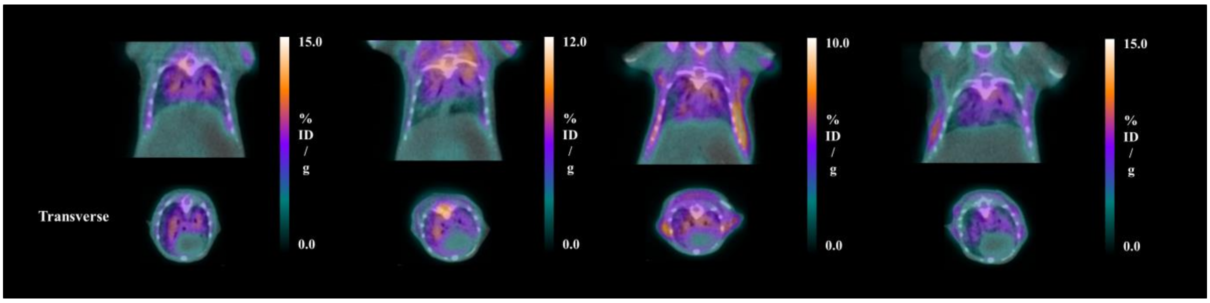

Supplementary Figure 11. MicroPET/CT fusion images of ^{18}F-FDG in mice with *A. fumigatus* lung infection 1 h after injection of ^{18}F-FDG (7.4 MBq).

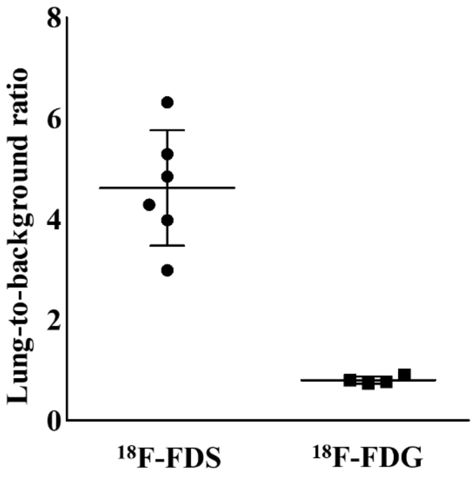

Supplementary Figure 12. Target (*A. fumigatus*-infected lung tissue)-to-background (normal muscle of hind leg) ratios of ^{18}F-FDS (2 h, n = 6) and ^{18}F-FDG (1 h, n = 4) post-injection (7.4 MBq). Data are expressed as the mean ± SD.

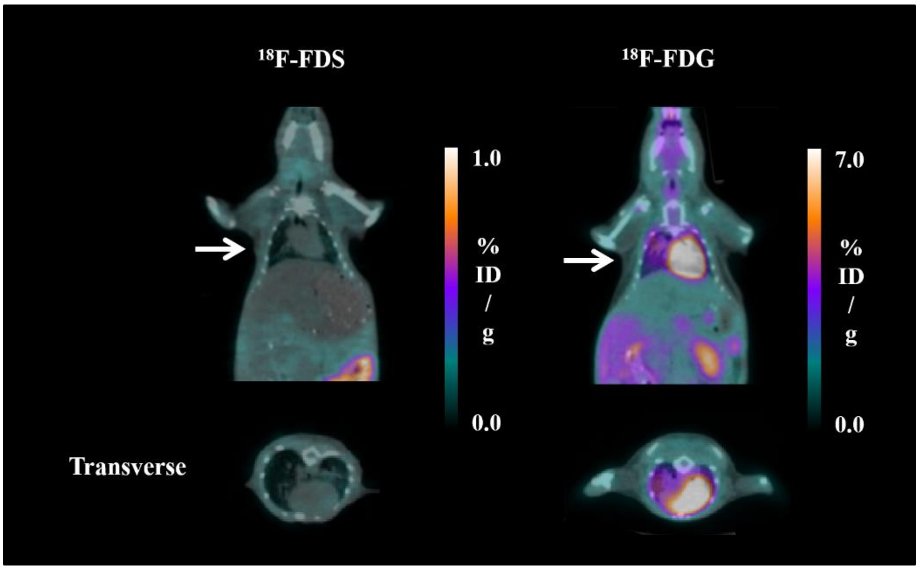

Supplementary Figure 13. MicroPET/CT fusion images of ^{18}F-FDS and ^{18}F-FDG in mice with B16F10 lung metastases (7.4 MBq).

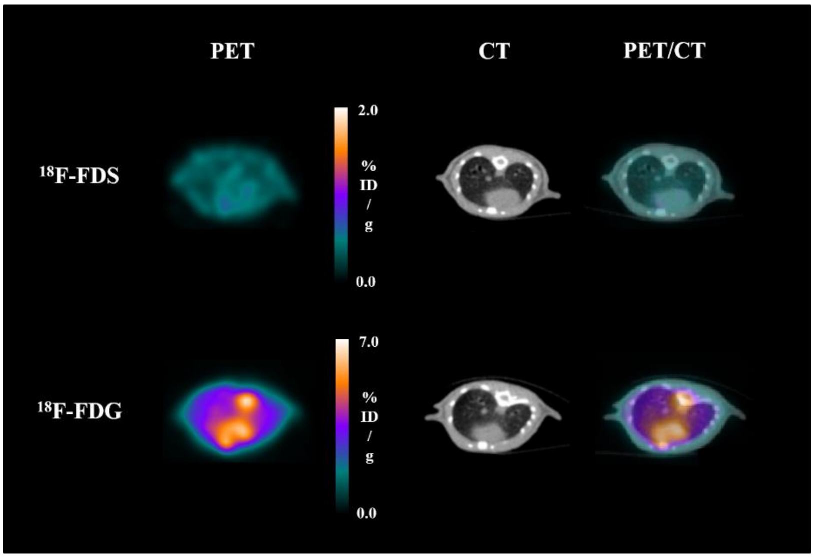

Supplementary Figure 14. MicroPET, CT, and PET/CT fusion images of ^{18}F-FDS and ^{18}F-FDG in mice with LPS-induced lung inflammation (7.4 MBq).

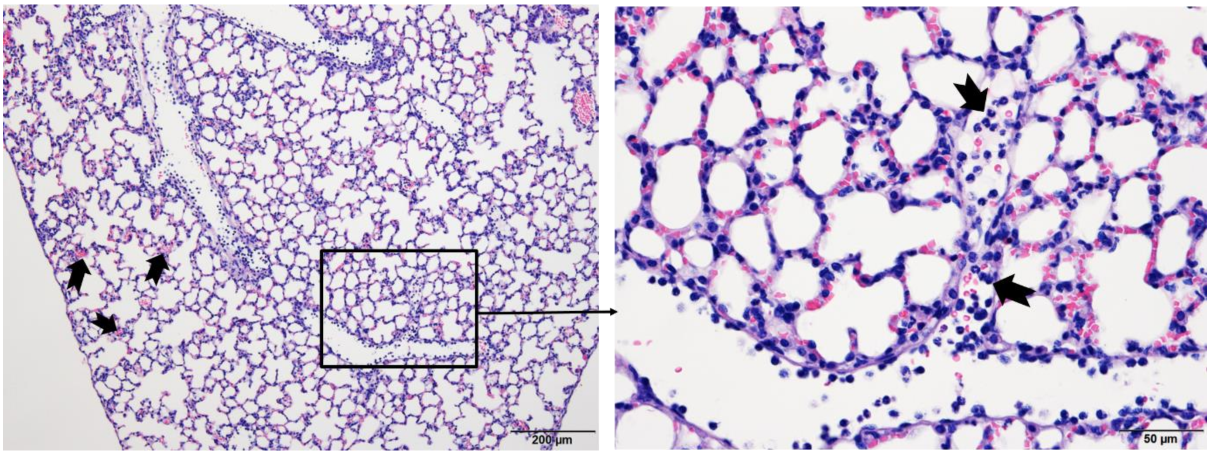

Supplementary Figure 15. Pathological examination of a mouse with LPS-induced acute lung injury (4 h after intraperitoneal LPS injection, n = 5). (Left) Hematoxylin and eosin (H&E) staining showing lung alveoli engorged with red blood cells (arrows) (original magnification × 100). (Right) Magnified view of the box in the left panel, showing the accumulation of neutrophils along the bronchioles.

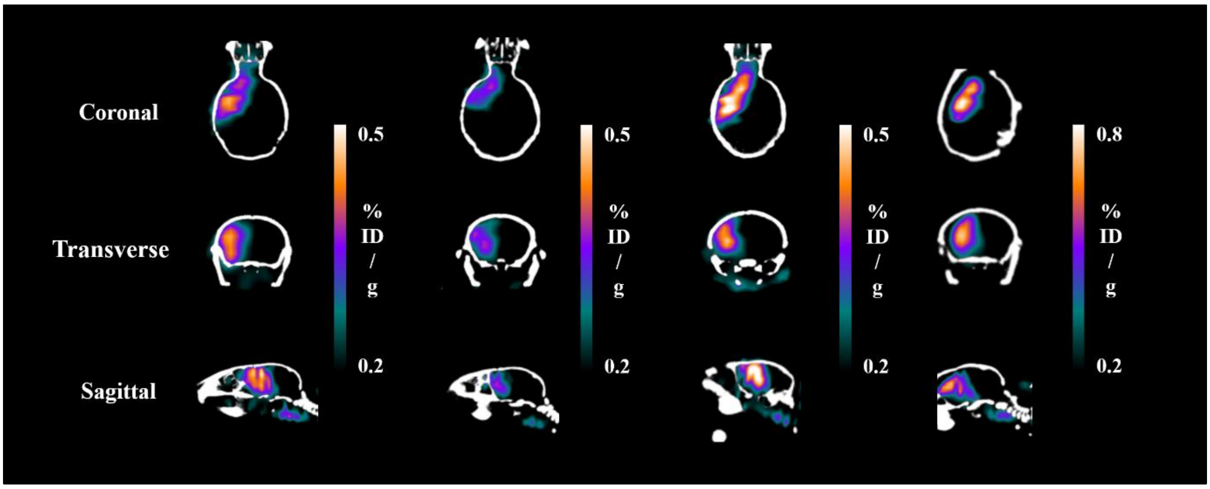

Supplementary Figure 16. MicroPET/CT fusion images of ^{18}F-FDS in mice with *A. fumigatus* brain infection at 48 h (7.4 MBq).

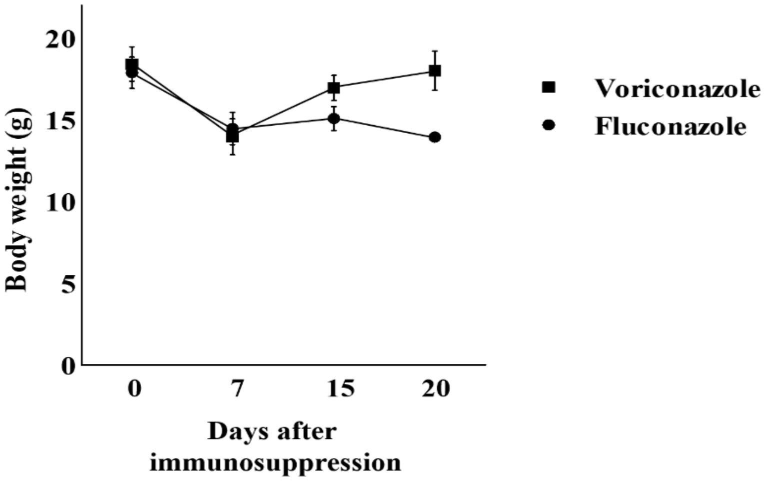

Supplementary Figure 17. Body weights of *A. fumigatus*-infected mice before and after treatment with voriconazole (n = 7) or fluconazole (0 and 7 days after treatment; n = 7, 15 days after treatment; n = 5, 20 days after treatment; n = 2) for up to 20 days. Data are expressed as the mean ± SD.
