# Supplementary material for: In vivo imaging of invasive aspergillosis with 18F-fluorodeoxysorbitol positron emission tomography
Source: Nat Commun. 2022 Apr 8;13:1926. doi: 10.1038/s41467-022-29553-5 (PMC8993802; doi:10.1038/s41467-022-29553-5)
Supplement: Supplementary file 4 — Description of Additional Supplementary Files [file 41467_2022_29553_MOESM4_ESM.pdf]

Title: Supplementary Movie 1.
Description: Dynamic ^{18}F-FDS PET images in mice with *A. fumigatus*-infected myositis for 2 h after *i.v.* injection of ^{18}F-FDS
